# Stakeholder perspectives towards the use of toxicants for managing wild pigs

**DOI:** 10.1371/journal.pone.0246457

**Published:** 2021-02-05

**Authors:** Ellary Tucker Williams, Christopher A. Lepczyk, Wayde Morse, Mark Smith

**Affiliations:** School of Forestry and Wildlife Sciences, Auburn University, Auburn, Alabama, United States of America; Sichuan University, CHINA

## Abstract

Wild pigs (*Sus scrofa*) are one of the most detrimental invasive mammals in the US. Lack of adequate population control has allowed pigs to become established across the landscape, causing significant ecological and economic damage. Given the need for additional tools for reducing wild pig populations, two toxicants, warfarin and sodium nitrite, are at the forefront of the discussion regarding future wild pig management. However, no research has examined stakeholders’ perspectives towards the use of toxicants in wild pig management. Given the lack of knowledge, our goal was to determine stakeholders’ perspectives towards the legal use of toxicants for managing wild pigs. We surveyed 1822 individuals from three stakeholder groups (hunters, farmers, and forestland owners) across Alabama during February 2018 using an online survey following the Tailored Design Method. All three stakeholder groups were generally supportive of toxicant use, though their views differed slightly by group. Furthermore, all stakeholder groups were supportive of toxicant purchasing and use regulations, while accidental water contamination, human health impact, and incorrect usage of a toxicant were stakeholders’ greatest concerns. These results indicate that these groups would likely be in support of using toxicants for wild pig management in Alabama and could be a model for other states or locations. Consequently, these results have direct implications for shaping policy and possible use of toxicants as a future wild pig management tool.

## Introduction

**Fig 1:**
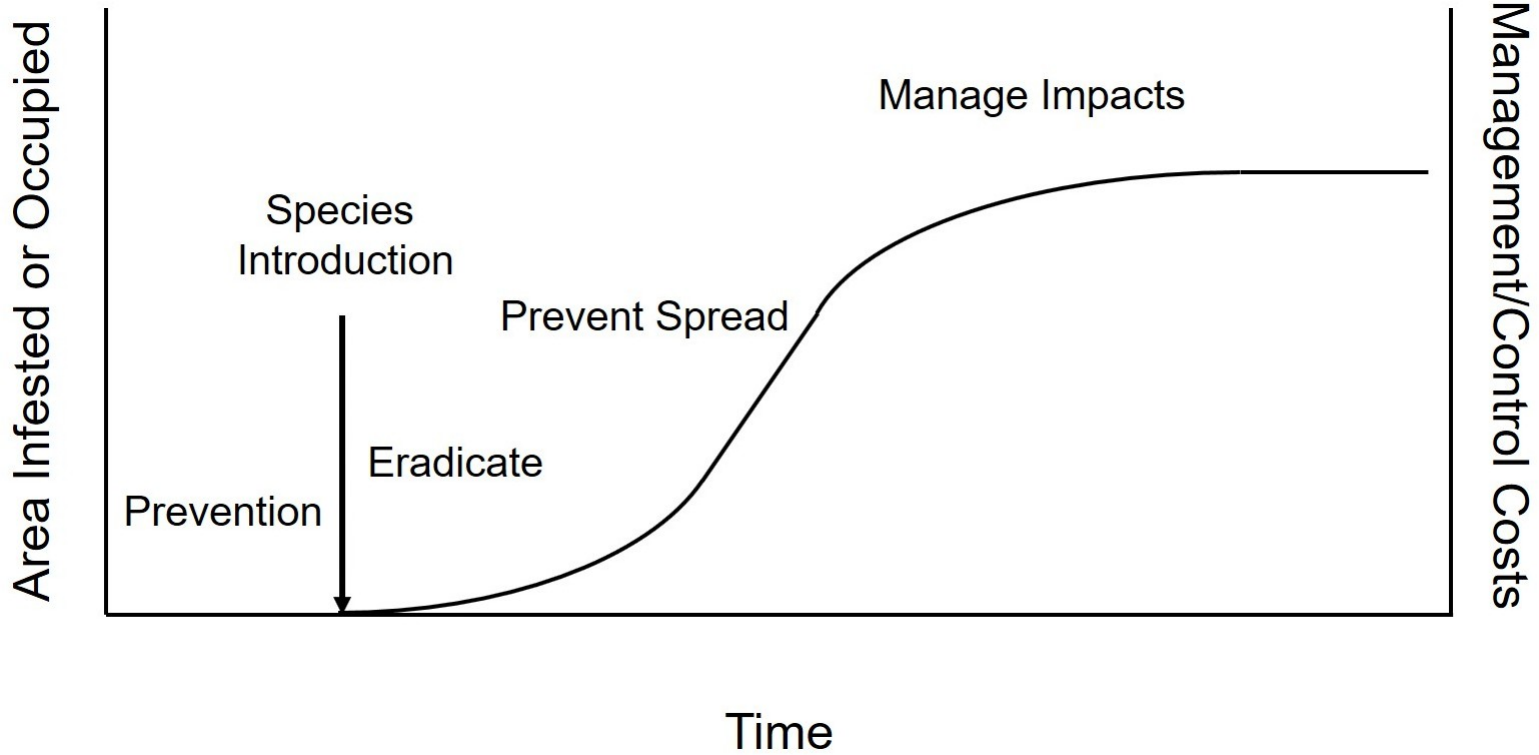
The invasion curve is a general model of invasion. Both costs and the area infested or occupied increase over time if a novel species is introduced and not eradicated. The four basic management considerations under this model are prevention, eradication, preventing spread, and managing impacts.

The United States has approximately 50,000 invasive species which are responsible for $128–131 billion of damage per year [1]. These cost estimates include both damages caused by invasive species and the costs associated with their management and control [2]. Due to novel disease exposure [3], competition, and/or predation, invasive species in the US are believed to be a major contributing factor for roughly half of the species listed as threatened or endangered under the Endangered Species Act [1, 3–6]. As a result, natural resource managers are using a variety of strategies to address the issue. While specific management techniques vary by species, the overall management of invasive species depends where on the invasion curve the species occurs [7–9; Fig 1]. One species that is near the end of the invasion curve and requires long-term management is the wild pig (*Sus scrofa*).

Native to Eurasia and Northern Africa [1, 10] and present on all continents, excluding Antarctica [11], wild pigs did not reach the continental US until the 1500s when early explorers (De Soto and Cortes) imported pigs as a food source [5, 10]. Beginning in the late 1900s, people facilitated the rapid expansion of wild pigs across the US, with little expansion due to natural dispersal [12–14]. Specifically, unlawful translocation of pigs to new areas with the intention of creating a new game species, animals escaping from fenced shooting preserves [10] or farms, and free-ranging pigs as an approach to husbandry [15] all contributed to the current distribution. Currently, wild pigs have been reported in 35 states making them the most abundant free-ranging ungulate ever introduced in the US [16]. Given their widespread distribution across the US, wild pigs have caused immense economic loss, particularly in the agricultural sector [17–22], damaged ecological systems [11, 14, 23–27], and posed a threat to public health [11, 22, 28–30]. Because of these economic and ecological impacts, wild pigs are a species of considerable management concern and a variety of management techniques have been developed to reduce their populations and associated impacts.

Wild pig management approaches include both lethal and non-lethal techniques. Lethal control techniques commonly include snares [31, 32], ground shooting/hunting (with or without dogs) [32, 33], aerial gunning [34], Judas pigs [35], and trapping (e.g., corral or box trap) followed by euthanasia [30, 32]. Non-lethal control techniques include fencing to limit exposure to sensitive areas [30, 36], repellents [37], and diversionary feeding [32]. In areas with established wild pig populations, management tends to focus on mitigating or reducing impact through lethal and non-lethal methods, whereas areas with small or recently established populations typically use eradication via trapping and shooting to stop establishment [12, 32, 38]. Either way, management is difficult due to the adaptive capability, evasiveness, and high fecundity of wild pigs. Furthermore, in most settings current management techniques have been ineffective, costly, and inefficient at reducing and maintaining wild pig population at acceptable levels [11, 32]. In incidences where wild pig eradication was successful, eradication efforts took years and were extremely costly [39–41]. As a result, researchers and managers have begun investigating the use of two orally delivered toxic baits containing warfarin or sodium nitrite as an additional tool available to control wild pig.

Reduction in wild pig populations using warfarin has been demonstrated in various study areas in Australia with population decreases varying between 67–99% [42–44]. Additionally, complete eradication was achieved on Santiago Island in the Galapagos through combined hunting efforts and warfarin use [45]. Warfarin competes with vitamin K in the synthesis of a protein, which is critical for the occurrence of blood-clotting. Therefore, wild pigs succumb to internal hemorrhaging from warfarin toxicosis [44]. Warfarin as a rodenticide has been registered in the United States since 1952 [46], however it was only recently approved by the United States Environmental Protection Agency (EPA) in 2017 for use on wild pigs (EPA Reg. No. 72500–26, Decision No. 510475). Texas was the only state to legalize its use, but due to threats of litigation from stakeholders, the products registration was withdrawn and subsequently is no longer available for use within the state [47].

Sodium nitrite is another toxicant that has demonstrated a high degree of potential for effectively reducing wild pig abundance [48–50]. Field testing of sodium nitrite in Australia showed reductions in wild pig populations varying between 63–89% [49], while pen trials in the US achieved 95% mortality [51]. Because wild pigs naturally lack the levels of methaemoglobin reductase necessary to counteract the effects of sodium nitrite [48], wild pigs expire due to severe methemoglobinemia, resulting in death from tissue hypoxia [51]. Sodium nitrite is currently registered for use in wild pig population control in New Zealand [50] and Australia and is conducting field trials in anticipation of making a submission for registration in the US [49, 51].

As research continues on the development of a toxicant for wild pigs in the US, understanding stakeholders’ knowledge and perspectives about the use of toxicants is critically important. In particular, the use of toxicants can be extremely controversial, and future use of toxicants as a population reduction tool will fail without stakeholder support. Whereas previous research has focused on understanding stakeholder attitudes towards wild pigs [18, 52–54], wild pig damage [18, 20, 55, 56] and management [20, 54, 55, 57–59], no research has evaluated stakeholder attitudes towards the use of toxicants for managing wild pigs in the US. In fact, to date only one study from Australia has evaluated stakeholder attitudes towards toxicant use in pigs [60]. Specifically, Koichi et al. [60] found that 34% of residents in Australia’s Wet Tropics World Heritage Area supported the use of toxicants [1080 (sodium fluoroacetate)] for managing wild pig populations, with reasons for opposition including lack of target specificity and humaneness.

Given that no studies have been conducted in the US, the overarching goal was to quantify stakeholder perspectives towards the use of toxicants for wild pig population control. Specifically, our objectives were to 1) determine stakeholder acceptability of sodium nitrite and warfarin for managing wild pig populations, 2) examine perspectives on various types of possible purchasing and use regulations, should toxicants be legalized for wild pigs, and 3) identify stakeholder concerns related to environmental and human health, application, and legal liability associated with the use of toxicants.

## Methods

To address the research objectives, we created an online social survey consisting of 58 questions, 23 of which were considered as part of this study. Of these 23 questions, eight pertained to attitudes towards wild pigs, toxicants and hypothetical toxicant use, and wild pig management approaches (S1 Appendix). The remaining 15 were sociodemographic questions that were used to describe the individuals who participated in the survey. Survey questions regarding respondents’ attitudes towards wild pigs addressed questions such as whether or not respondents felt positively or negatively about the presence of wild pigs, and how respondents would like to see wild pig population changed in the future. Before respondents were able to answer any questions pertaining to toxicant use in wild pig management, they were provided with a list of pertinent information about both warfarin and sodium nitrite (S1 Appendix). Such information was provided in order to ensure that each respondent had the equivalent baseline knowledge and understanding of the two toxicants being considered. Questions regarding toxicant use addressed stakeholders’ level of acceptability, concerns surrounding the use of toxicants, and various hypothetical purchasing and use regulation, should a toxicant be registered for use. The remaining questions addressed respondent perspectives on future wild pig management objectives. Previous surveys on stakeholder perspectives towards wild pigs provided insight for survey questions and formatting [e.g., 18, 61, 62].

The survey instrument was designed to be disseminated to three key stakeholder groups, hunters, farmers, and forestland owners throughout Alabama. These three groups were selected because they own the majority of private land within Alabama [63, 64] and are most likely to interact with and be affected by wild pigs in the state. The draft survey was peer-reviewed in a pilot study of 10 volunteers from the School of Forestry and Wildlife Sciences at Auburn University, and reviewed by the Alabama Farmers Federation (ALFA), and Alabama Forest Owners Association (AFOA) to improve the quality of the survey instrument. The final survey was approved by the Auburn University Institutional Review Board (IRB) (Protocol #17–397 EX 1710).

Following the Tailored Design Method [65], we administered the survey via the Internet in January, 2018, using Qualtrics. An invitation email with the link to the survey was disseminated to each of the three stakeholder groups using email addresses of the group members, followed by two reminder emails at two and four weeks after the initial email. Specifically, emails were sent by ALFA to all Alabama row crop, produce, hay, cattle, domestic pig, poultry, and sheep farmers within the ALFA membership list, which equated to approximately 10,700 individuals. To survey hunters we purchased 5,000 email addresses of individuals who had purchased an Alabama hunting license for the 2017–2018 season from the Alabama Department of Conservation and Natural Resources (ADCNR). However, only 4,621 of the 5,000 email addresses were valid due to duplicates and obsolete email addresses. Finally, the AFOA distributed the email to all associated members who owned forestland in Alabama, approximately 4,000 individuals. In total approximately 19,321 people received the invitation to participate in the survey. To differentiate between stakeholders each group received a separate and unique online link to the survey.

All survey respondents were required to acknowledge that they had read the consent letter in order to gain access to the survey, thereby verifying that they were at least 19 years of age and agreeing to participate in the research project. At the end of the survey individuals were given the opportunity to provide their email address if they wanted to receive a summary of the survey results. To increase response rates, the survey was incentivized. At the completion of the survey, individuals were given the option to submit their name and mailing address in a prize drawing to win 1 of 5 Amazon gift cards, each valued at $100. The survey was closed at the beginning of March 2018 and winners were awarded.

Aside from demographic questions used to describe the sample, the remaining survey questions were scored on a Likert-type scale with the majority being either five, seven, or eight points. Only one question used an eight point question, which was the result of adding an eighth option of eradiation to the seven point survey question pertaining to how stakeholders would like to see future wild pig populations change (S1 Appendix). We used these different scales sizes in attempts to gain a more nuanced understanding of stakeholder perspectives to certain questions. Notably, while different questions contained different possible numbers of answers, they are only being compared between stakeholder groups and not across different questions.

Initial statistical analysis consisted of descriptive statistics of all questions. An important note, not all respondents were required to answer all questions in the survey therefore response rate varied by question. To determine if perspectives towards wild pigs and the use of toxicants in wild pig management differed between hunters, forestland owners, and farmers, we used a one-way ANOVA. If differences were found, we used a Tukey post-hoc test to determine which groups differed from one another. Due to the large sample size associated with each question, we used a univariate general linear model to estimate the regression coefficients (betas) of each stakeholder group per survey question. Due to the large sample size, the betas were used to examine effect size and if differences identified by the ANOVA analysis had any subject-matter significance [66]. Beta values ≥ 0.5 were considered important as they equated to a ½ point change on the Likert scale. Results are presented as means ± standard deviation, with general linear model results presented as beta, confidence interval, and p-value. All statistical analysis were conducted in accordance with Vaske [67] using SPSS 24 [68] with p-value ≤ 0.05 considered significant.

A total of 1822 (~9%) individuals responded to the survey, however response rates varied by stakeholder group. A total of 668 hunters, 1055 farmers, and 99 forestland owners responded, equating to a 14%, 10%, and 2% response rate, respectively. The low response rate of forestland owners compared to farmers and hunters was due to break in survey method. Specifically, AFOA did not send a specific email inviting members to participate in the survey and instead included the survey invitation and link as part of a general email that also contained additional information associated with the AFOA. Therefore, AFOA members likely did not notice the survey option within the body of the email. However, despite the low sample size, forestland owner responses were similar to the other two groups, therefore they are included in the analyses.

## Results

### Demographics

Of survey respondents, the majority were Caucasian males who were 50 years of age or older and had lived in Alabama for approximately the same number of years (Table 1). In regard to household income, 87% (n = 1,383) of respondents earned between $50,000 and greater than $150,000 in 2017 (Table 1). Additionally, 67% (n = 1,466) of respondents had some form of higher education (Table 1) and most owned land in Alabama (91%, n = 1,347) with 65% (n = 1,321) of owned land varying in size between less than 50 acres and 200 acres (Table 1). In regards to the primary purpose of owning land, farming (30%, n = 1,348), forest products or timber (27%, n = 1348), and residential (27%, n = 1,348) were the most commonly selected responses while 66% of respondents indicated that they lived on their property (n = 1,347; Table 1). Respondents lived in urban, suburban, and rural communities, with ~29% living in a town or city with many neighbors, ~28% living in an area outside of a town with scattered neighbors, and ~43% living in a rural area with few neighbors (n = 1,705, Table 1). Respondents were from every county in Alabama, with Baldwin, Mobile, Jefferson, and Tuscaloosa County having the greatest number of respondents.

**Table 1:** The percentage of and number (n) of all respondents who answered for each sociodemographic factor across the three Alabama stakeholder groups.

Sociodemographic Factors	Variable	% (n)
Gender	Male	90.7% (1334)
	Female	9.3% (136)
Highest Level of Education	Some high school	1.3% (19)
	High School/GED	12.6% (184)
	Some college, but no degree	19.4% (285)
	Vocational/professional certification	6.9% (101)
	Associates	7.7% (113)
	Bachelor’s Degree	31.2% (457)
	Master’s degree	15.1% (222)
	Doctorate	5.8% (85)
Age (years)	20–29	1.5% (22)
	30–39	4.7% (68)
	40–49	7.8% (113)
	50–59	35.8% (519)
	60–69	33.9% (491)
	70–79	14.2% (206)
	80–89	1.9% (28)
	90–99	0.1% (2)
Ethnicity	African American	1.5% (22)
	Caucasian	95.3% (1388)
	Chinese	0.1% (1)
	Latino	0.1% (2)
	Native American	2% (29)
	Other	1% (15)
Household Income 2017	< $14,999	0.9% (13)
	$15,000-$19,999	0.4% (6)
	$20,000-$24,999	1.4% (20)
	$25,000-$34,999	3.2% (44)
	$35,000-$49,000	6.9% (96)
	$50,000-$74,000	18.8% (260)
	$75,000-$99,999	19% (263)
	$100,000-$149,999	26.9% (372)
	$150,000 or more	22.4% (309)
Community Type	Town/city with many neighbors	29.3% (499)
	Outside a town with scattered neighbors	27.6% (470)
	Rural area with few neighbors	43.2% (736)
Years lived in Alabama	1–10	0.6% (9)
	11–19	1.1% (17)
	20–29	4.1% (66)
	30–39	10.7% (171)
	40–49	13.7% (218)
	50–59	35.3% (564)
	60–69	25.4% (405)
	70–79	8.1% (130)
	≥ 80	1.1% (17)

### Attitudes towards wild pigs

Despite significant differences found between groups, all stakeholder groups indicated an attitude of “dislike” for wild pigs (1.8 ± 1.2, Table 2). Specifically, all groups wanted to see a declining wild pig population trend (1.9 ± 1.2, Table 2). In terms of the hypothetical wild pig management objectives presented to survey respondents, “decreasing wild pig populations within the state” (6.2 ± 1.3), “reducing wild pig damage” (5.8 ± 1.1), and “increasing research to develop more cost and time effective control strategies” (5.4 ± 1.5, Table 3) were of greatest priority to all groups. Farmers, forestland owners and hunters differed significantly regarding the priority level they designated for “decreasing wild pig populations within the state,” with hunters being significantly lower than the other two groups and forestland owners being significantly greater (Table 3). Hunters deemed “reduce wild pig damage” and “increase research to develop more cost and time effective control strategies” as significantly less of a priority than farmers and forestland owners (Table 3).

**Table 2:** Summary statistics by stakeholder group of key questions pertaining to stakeholder attitudes towards and future population levels of wild pigs in Alabama.

				Hunter				Farmer				Forestland Owner			
Question	Grand Mean ± SD (n)	F_(df)_	P-value	Mean ± SD (n)	Beta^d^	CI	Partial p-value	Mean ± SD (n)	Beta^e^	CI	Partial p-value	Mean ± SD (n)	Beta^f^	CI	Partial p-value
Attitude towards wild pigs^a^	1.8 ± 1.2 (1,700)	48.37_(2, 1697)_	<0.01^1^^2^^3^	2.1 ± 1.5 (629)	0.54	0.12	<0.01	1.6 ± 1.0 (979)	0.34	0.26	0.01	1.2 ± 0.6 (92)	-0.88	0.27	<0.01
Expressed future wild pig population trend^b^	1.9 ± 1.2 (1,519)	58.62_(2, 1516)_	<0.01^1^^3^	2.3 ± 1.4 (555)	0.62	0.12	<0.01	1.7 ± 1.0 (879)	0.26	0.26	0.04	1.5 ± 0.8 (85)	-0.88	0.26	<0.01
Importance of developing a management plan to meet the above stated future wild pig population trend^c^	3.9 ± 1.4 (1,520)	2.51_(2, 1517)_	0.08	3.8 ± 1.3 (554)	-0.12	0.15	0.093	3.94 ± 1.4 (881)	-0.18	0.31	0.25	4.1 ± 1.4 (85)	0.30	0.32	0.06

a = 7 point Likert scale (1 = I dislike wild pigs, 4 = Neutral, 7 = I like wild pigs), b = 8 point Likert scale (1 = Completely eradicate, 5 = Stay the same, 8 = Increase drastically), c = 5 point Likert scale (1 = Extremely unimportant, 3 = Neutral, 5 = Extremely important). Beta; d = Farmers are the reference variable, e = Forestland owners are the reference variable, f = Hunters are the reference variable. 1 = hunters and forestland owners significantly differ, 2 = forestland owners and farmers significantly differ, 3 = farmers and hunters significantly differ.

**Table 3:** Summary statistics by stakeholder group of survey questions relating to the priority level assigned to hypothetical wild pig management objectives.

				Hunter				Farmer				Forestland Owner			
Question	Grand Mean ± SD (n)	F_(df)_	P-value	Mean ± SD (n)	Beta^a^	CI	Partial p-value	Mean ± SD (n)	Beta^b^	CI	Partial p-value	Mean ± SD (n)	Beta^c^	CI	Partial p-value
Priority level of decreasing wild pig populations within the state	6.2 ± 1.3 (1,467)	41.46_(2, 1464)_	<0.01^1^^2^^3^	5.8 ± 1.4 (537)	-0.53	0.13	<0.01	6.4 ± 1.1 (849)	-0.41	0.28	<0.01	6.8 ± 0.6 (81)	0.94	0.29	<0.01
Priority level of reducing wild pig damage	5.8 ± 1.1 (1,470)	42.31_(2 1467)_	<0.01^1^^3^	5.5 ± 1.3 (538)	-0.50	0.12	<0.01	6.0 ± 1.0 (851)	-0.33	0.26	0.01	6.3 ± 0.9 (81)	0.83	0.26	<0.01
Priority level of increasing research to develop more cost and time effective control strategies	5.4 ± 1.5 (1,467)	18.63_(2, 1464)_	<0.01^1^^3^	5.1 ± 1.5 (536)	-0.42	0.16	<0.01	5.5 ± 1.4 (850)	-0.33	0.22	0.05	5.9 ± 1.3 (81)	0.75	0.34	<0.01
Priority level of making high tech equipment (e.g., cell phone monitored trapping equipment) available to rent to landowners at a reasonable cost	5.2 ± 1.5 (1,459)	14.43_(2, 1956)_	<0.01^1^^3^	5.0 ± 1.6 (533)	-0.40	0.16	<0.01	5.4 ± 1.3 (847)	-0.15	0.34	0.37	5.5 ± 1.4 (79)	0.56	0.35	<0.01
Priority level of restoring damaged ecosystems	5.1 ± 1.3 (1,461)	2.50_(2, 1458)_	0.08	5.0 ± 1.4 (532)	-0.17	0.15	0.03	5.1 ± 1.3 (848)	0.07	0.31	0.67	5.1 ± 1.3 (81)	0.10	0.32	0.54
Priority level of stronger enforcement of current regulation and policy	5.1 ± 1.8 (1,469)	31.52_(2, 1466)_	<0.01^1^^3^	4.6 ± 1.8 (536)	-0.74	0.19	<0.01	5.3 ± 1.7 (852)	-0.07	0.40	0.74	5.4 ± 1.8 (81)	0.81	0.41	<0.01
Priority level of creating wild pig management cooperatives to reduce individual cost and labor demands in order to remove wild pigs from larger areas of land	5.0 ± 1.4 (1,465)	6.97_(2, 1462)_	<0.01^3^	4.8 ± 1.5 (535)	-0.28	0.15	<0.01	5.1 ± 1.3 (849)	0.26	0.33	0.11	4.9 ± 1.4 (81)	0.02	0.33	0.91
Priority level of increasing funding to better facilitate state management	4.9 ± 1.5 (1,459)	12.13_(2, 1456)_	<0.01^3^	4.6 ± 1.6 (533)	-0.41	0.17	<0.01	5.0 ± 1.5 (846)	-0.02	0.36	0.91	5.1 ± 1.4 (80)	0.43	0.36	0.02
Priority level of creating a financial assistance program that aims to compensate individuals for economic loss associated with wild pig damage	4.4 ± 1.7 (1,462)	9.13_(2, 1459)_	<0.01^3^	4.1 ± 1.8 (534)	-0.40	0.19	<0.01	4.5 ± 1.7 (847)	0.05	0.39	0.81	4.5 ± 1.5 (81)	0.35	0.40	0.08
Priority level of making recreational wild pig hunting illegal	1.9 ± 1.7 (1,456)	15.23_(2, 1453)_	<0.01^3^	1.6 ± 1.5 (533)	-0.5	0.18	<0.01	2.1 ± 1.7 (844)	0.28	0.38	0.15	1.8 ± 1.6 (79)	0.22	0.39	0.27
Priority level of increasing wild pig populations within the state	1.5 ± 1.2 (1,468)	18.64_(2, 1465)_	<0.01^1^^3^	1.7 ± 1.3 (536)	0.36	0.13	<0.01	1.4 ± 1.1 (851)	0.19	0.27	0.16	1.2 ± 0.9 (81)	-0.55	0.28	<0.01

Beta; a = Farmers are the reference variable, b = Forestland owners are the reference variable, c = Hunters are the reference variable. 1 = hunters and forestland owners significantly differ, 2 = forestland owners and farmers significantly differ, 3 = farmers and hunters significantly differ.

All questions are based on 7-point Likert scale (1 = Very low priority, 4 = Neutral, 7 = Very high priority).

### Toxicants

Sodium nitrite (3.9 ± 1.4) was found to be more acceptable than warfarin (2.8 ± 1.5, Table 4) as a method to control wild pig populations. However all groups significantly differed in their acceptability of using sodium nitrite as a method for wild pig population control. Hunters showed significantly lower acceptability than farmers and forestland owners while forestland owners showed significantly greater acceptability than the other two groups. Hunters differed significantly from farmers and forestland owners regarding the acceptability of warfarin with a lower level of acceptability (Table 4).

**Table 4:** Summary statistics by stakeholder group of survey questions pertaining to the level of acceptability for toxicant use in wild pig management.

				Hunter				Farmer				Forestland Owner			
Question	Grand Mean ± SD (n)	F_(df)_	P-value	Mean ± SD (n)	Beta^a^	CI	Partial p-value	Mean ± SD (n)	Beta^b^	CI	Partial p-value	Mean ± SD (n)	Beta^c^	CI	Partial p-value
Acceptability of sodium nitrite as a method of wild pig population control	3.9 ± 1.4 (1,577)	33.23_(2, 1574)_	<0.01^1^^2^^3^	3.50 ± 1.5 (585)	-0.50	0.15	<0.01	4.0 ± 1.3 (906)	-0.49	0.31	<0.01	4.5 ± 1.0 (86)	1.00	0.32	<0.01
Acceptability of warfarin as a method of wild pig population control	2.8 ± 1.5 (1,576)	10.58_(2, 1573)_	<0.01^1^^3^	2.6 ± 1.6 (584)	-0.34	0.16	<0.01	2.9 ± 1.5 (905)	-0.19	0.35	0.28	3.1 ± 1.6 (87)	0.53	0.36	<0.01

Beta; a = Farmers are the reference variable, b = Forestland owners are the reference variable, c = Hunters are the reference variable. 1 = hunters and forestland owners significantly differ, 2 = forestland owners and farmers significantly differ, 3 = farmers and hunters significantly differ.

All questions are based on 5-point Likert scale (1 = Completely unacceptable, 3 = Neutral, 5 = Completely acceptable).

In regards to various hypothetical purchasing and use regulations, all groups showed support for four of the five options presented in the survey (Table 5). Hunters showed significantly lower levels of support for an individual being “19 years of age or older to purchase a toxicant” (3.8 ± 1.6), and the “toxic bait and bait dispenser being required by law to be sold together to limit access by non-target species” (3.7 ± 1.5) than farmers and forestland owners (Table 5). Additionally hunters showed significantly lower support for a toxicant only being sold by licensed vendors (3.7 ± 1.5), and requiring an individual to obtain a use permit by completing an online training course in toxicant application and safety before being allowed to purchase a wild pig toxicant (3.8 ± 1.5) than farmers (Table 5).

**Table 5:** Summary statistics by stakeholder group of survey questions regarding the level of support for hypothetical purchasing and use regulations, if a toxicant were legalized for wild pig management.

				Hunter				Farmer				Forestland Owner			
Question	Grand Mean ± SD (n)	F_(df)_	P-value	Mean ± SD (n)	Beta^a^	CI	Partial p-value	Mean ± SD (n)	Beta^b^	CI	Partial p-value	Mean ± SD (n)	Beta^c^	CI	Partial p-value
19 years old to purchase toxicant	4.1 ± 1.4 (1,560)	21.85_(2, 1557)_	<0.01^1^^3^	3.8 ± 1.58 (577)	-0.45	0.15	<0.01	4.2 ± 1.3 (898)	-0.23	0.31	0.14	4.5 ± 1.1 (85)	0.68	0.32	<0.01
Toxic bait and bait dispenser required to be sold together	4.0 ± 1.4 (1,549)	16.75_(2, 1546)_	<0.01^1^^3^	3.7 ± 1.5 (575)	-0.41	0.14	<0.01	4.1 ± 1.2 (889)	-0.01	0.30	0.93	4.1 ± 1.2 (85)	0.42	0.31	<0.01
Only sold by licensed vendors	3.9 ± 1.4 (1,549)	8.96_(2, 1546)_	<0.01^3^	3.7 ± 1.5 (577)	-0.31	0.15	<0.01	4.1 ± 1.3 (887)	0.06	0.32	0.68	4.0 ± 1.3 (85)	0.25	0.32	0.12
Required to obtain a purchase and use permit through an online training course	3.9 ± 1.4 (1,546)	4.08_(2, 1543)_	0.02^3^	3.8 ± 1.5 (573)	-0.21	0.15	<0.01	4.0 ± 1.3 (888)	0.12	0.31	0.43	3.9 ± 1.3 (85)	0.09	0.32	0.59
Not available to the public, only agency personnel have access to toxicant and are legally allowed to use it	2.9 ± 1.5 (1,547)	0.80_(2, 1544)_	0.45	2.8 ± 1.5 (574)	-0.08	0.16	0.31	2.9 ± 1.5 (888)	0.16	0.35	0.35	2.8 ± 1.5 (85)	-0.08	0.36	0.66

Beta; a = farmers are the reference variable, b = forestland owners are the reference variable, c = hunters are the reference variable. 1 = hunters and forestland owners significantly differ, 2 = forestland owners and farmers significantly differ, 3 = farmers and hunters significantly differ.

All questions are based on 5-point Likert scale (1 = Do not support at all, 3 = Neutral, 5 = Completely support).

Regarding the level of concern in relation to any toxicant use as a method of wild pig population control in Alabama, “accidental water contamination” (4.3 ± 0.9), “human health impact” (4.3 ± 1.0), and “incorrect usage of a toxicant” (4.2 ± 1.0) were of highest concern for all groups (Table 6). Amongst the top three concerns, only “incorrect usage of a toxicant” significantly differed between stakeholder groups (Table 6). When looking at the collective list of concerns, farmers were significantly less concerned about “incorrect usage of a toxicant” (4.20 ± 0.96), “eradicating wild pigs entirely” throughout the state (2.6 ± 1.6), and “public opinion” about a toxicant (2.8 ± 1.3) than hunters (Table 5). While hunters were significantly more concerned about the “personal financial cost” associated with a toxicant (3.0 ± 1.2) and “eradicating wild pigs entirely” (2.9 ± 1.5) than forestland owners (Table 6).

**Table 6:** Summary statistics by stakeholder group of key survey questions pertaining to the level of concern with any toxicant use in wild pig management.

				Hunter				Farmer				Forestland Owner			
Question	Grand Mean ± SD (n)	F_(df)_	P-value	Mean ± SD (n)	Beta^a^	CI	Partial p-value	Mean ± SD (n)	Beta^b^	CI	Partial p-value	Mean ± SD (n)	Beta^c^	CI	Partial p-value
Accidental water contamination concern	4.3 ± 0.9 (1,526)	2.76_(2, 1523)_	0.06	4.4 ± 0.9 (558)	0.12	0.10	0.02	4.2 ± 0.9 (882)	0.02	0.21	0.87	4.2 ± 1.0 (86)	-0.13	0.22	0.22
Human health impact concern	4.3 ± 1.0 (1,522)	1.55_(2, 1519)_	0.21	4.3 ± 1.0 (557)	0.09	0.11	0.08	4.2 ± 1.0 (879)	-0.06	0.23	0.58	4.3 ± 1.0 (86)	-0.03	0.23	0.78
Incorrect usage of a toxicant concern	4.2 ± 1.0 (1,524)	4.60_(2, 1521)_	0.01^3^	4.3 ± 0.9 (557)	0.14	0.10	<0.01	4.2 ± 1.0 (881)	0.07	0.21	0.52	4.1 ± 1.0 (86)	-0.21	0.22	0.05
Legal liability for non-target damage (e.g., accidental death of livestock) concern	4.2 ± 1.0 (1,521)	2.54_(2, 1518)_	0.08	4.2 ± 1.0 (556)	0.11	0.11	0.03	4.1 ± 1.0 (880)	0.04	0.22	0.73	4.1 ± 1.0 (85)	-0.15	0.23	0.18
Impact on non-target species concern	4.2 ± 1.1 (1,535)	0.51_(2, 1532)_	0.60	4.2 ± 1.08 (563)	0.05	0.11	0.40	4.2 ± 1.0 (886)	0.05	0.24	0.70	4.1 ± 1.1 (86)	-0.09	0.24	0.44
Accidental soil contamination concern	4.1 ± 1.1 (1,527)	2.63_(2, 1524)_	0.07	4.2 ± 1.1 (560)	0.12	0.11	0.03	4.1 ± 1.0 (882)	0.06	0.24	0.64	4.0 ± 1.2 (85)	-0.18	0.24	0.15
Ability to regulate use of a toxicant concern	4.0 ± 1.0 (1,525)	2.26_(2, 1522)_	0.10	4.0 ± 1.0 (556)	0.10	0.11	0.08	4.0 ± 1.0 (883)	0.10	0.23	0.41	3.9 ± 1.1 (86)	-0.20	0.24	0.10
Effectiveness of toxicant concern	4.0 ± 1.0 (1,521)	2.23_(2, 1518)_	0.11	4.0 ± 1.1 (556)	-0.11	0.11	0.06	4.1 ± 1.00 (879)	0.15	0.24	0.20	3.9 ± 1.1 (86)	-0.05	0.24	0.70
Personal financial cost concern	3.4 ± 1.1 (1,522)	1.14_(2, 1519)_	0.32	3.3 ± 1.1 (557)	-0.05	0.12	0.37	3.4 ± 1.1 (880)	0.17	0.25	0.18	3.2 ± 1.2 (85)	-0.12	0.26	0.37
Personal time requirement concern	2.9 ± 1.1 (1,520)	3.14_(2, 1517)_	0.04^1^	3.0 ± 1.2 (556)	0.07	0.12	0.24	2.9 ± 1.1 (879)	0.25	0.26	0.05	2.7 ± 1.1 (85)	-0.32	0.26	0.01
Humaneness concern	2.9 ± 1.5 (1,533)	1.89_(2, 1530)_	0.15	3.0 ± 1.5 (561)	0.14	0.16	0.07	2.8 ± 1.5 (886)	0.06	0.33	0.71	2.8 ± 1.5 (86)	-0.21	0.34	0.23
Public opinion concern	2.8 ± 1.4 (1,504)	6.71_(2, 1501)_	0.00^3^	3.0 ± 1.4 (548)	0.26	0.15	<0.01	2.8 ± 1.3 (871)	0.09	0.31	0.56	2.7 ± 1.3 (85)	-0.35	0.32	0.03
Eradicating wild pigs entirely concern	2.7 ± 1.6 (1524)	9.15_(2, 1521)_	0.00^1^^3^	2.9 ± 1.5 (558)	0.28	0.17	<0.01	2.6 ± 1.6 (882)	0.34	0.35	0.05	2.3 ± 1.6 (84)	-0.63	0.36	<0.01

Beta; a = Farmers are the reference variable, b = Forestland owners are the reference variable, c = Hunters are the reference variable. 1 = hunters and forestland owners significantly differ, 2 = forestland owners and farmers significantly differ, 3 = farmers and hunters significantly differ.

All questions are based on 5-point Likert Scale (1 = Totally unconcerned, 3 = Neutral, 5 = Extremely concerned).

While the direction and relative scores were similar, a total of 51 significant differences were found between stakeholder groups among the 11 questions and associated sub-questions analyzed, excluding socio-demographic questions. Of those 51 significant differences found, 19 occurred between hunters and forestland owners (~37%), 4 occurred between forestland owners and farmers (~ 8%), and 28 occurred between farmers and hunters (~55%, Tables 2–6). Due to the questionable nature of claiming our samples as representative of these stakeholder groups, the differences that were identified suggests further exploration may be warranted.

### Univariate analysis

Approximately 17% (n = 126) of the betas were considered noteworthy with a value of > ± 0.50 of a Likert point. Of that 17%, roughly 55% occurred between forestland owners and hunters while the remaining 45% occurred between hunters and farmers. On a seven point Likert scale, hunters had 0.54 (± 0.12) more positive attitude towards wild pigs than farmers whereas forestland owners had -0.88 (± 0.27) more negative attitude towards wild pigs than hunters (Table 2). Hunters had 0.62 (± 0.12) more positive desire for increasing future wild pig population trend than farmers, whereas forestland owners had a -0.88 (± 0.26) desire for decreasing future wild pig population trends than hunters on an eight point Likert scale (Table 2).

On a five point Likert scale, hunters had -0.5 (± 0.15) less acceptability of sodium nitrite as a method of wild pig population control than farmers. On the same five point scale, forestland owners had 1.00 (± 0.32) greater acceptability of sodium nitrite as a method of wild pig population control than hunters. Forestland owners had 0.53 (± 0.36) greater acceptability of warfarin as a method of wild pig population control than hunters. Additionally, forestland owners had 0.86 (± 0.32) more support for requiring an individual to be 19 years of age or older to be able to purchase a toxicant than hunters. And forestland owners had -0.63 (± 0.36) less concern for eradicating wild pigs entirely than hunters (Table 3), again on a five point Likert scale.

Furthermore, hunters designated reducing wild pigs damage as -0.50 (± 0.12) less of a priority than farmers, while forestland owners designated reducing wild pig damage as 0.83 (± 0.26) greater of a priority than hunters. Additionally, forestland owners identified increasing wild pig populations within the state as -0.55 (± 0.28) less of a priority than hunters. Hunters showed -0.53 (± 0.13) greater priority for decreasing wild pig populations within the state than farmers while forestland owner showed 0.94 (± 0.29) greater priority for decreasing wild pig populations within the state than hunters. Hunters designated having stronger enforcement of current wild pig policy and regulations as -0.74 (± 0.19) less of a priority than farmers. Contrastingly, forestland owners deemed having stronger enforcement of current wild pig policy and regulation as 0.81 (± 0.41) greater of a priority than hunters. Forestland owners identified increasing research to develop more cost and time effective wild pig control strategies as 0.75 (± 0.34) greater priority than hunters. Furthermore, making high tech wild pig trapping equipment available to rent to landowners at a reasonable cost was designated as 0.56 (± 0.35) greater priority to forestland owners than hunters. Hunters deemed making recreational wild pig hunting illegal as -0.5 (± 0.18) less of a priority than farmers (Table 3). All questions regarding stakeholder priority of management objectives was on a seven point Likert scale.

## Discussion

Overall, the majority of stakeholders dislike wild pigs and want to see reductions of wild pig populations on the landscape. Furthermore, decreasing wild pig population, reducing damage associated with wild pigs, and increasing research to develop more time and cost effective wild pig management strategies were deemed top priority objectives by all groups. Stakeholder groups supported the use of both toxicants, with greater support for sodium nitrite than warfarin. All stakeholder groups were generally supportive of the various purchasing and use regulations presented in the survey, however they were least supportive of toxicants being unavailable to the public for use. Finally, accidental water contamination, human health impact, and incorrect usage of a toxicant were identified as top concerns for all groups.

Despite being significantly different statistically, all stakeholders perspectives towards wild pigs and wild pig management that were similar to one another in terms of social meaning. All groups expressed a general dislike for wild pigs were in agreement that decreasing wild pig populations in Alabama was a high to very high priority management objective. Additionally, all groups wanted to see future wild pig populations drastically decreased on the landscape. These findings support previous research [53, 54, 57] that farmers and landowners believed wild pig populations should be significantly reduced or eradicated whenever possible However, the hunting stakeholder group in our study wanted a less drastic decrease in wild pig populations than forestland owners and farmers.

Sodium nitrite was found to be acceptable by stakeholders, while warfarin was neutrally to somewhat unacceptable in wild pig management. Acceptability of sodium nitrite in our study was much greater than that found by Koichi et al. [60] regarding general toxicant use, while acceptability of warfarin was only slightly higher in comparison. Similar surveys assessing stakeholder acceptability of toxicant use in invasive species management had comparable findings. Specifically, Fisher et al. [69] found that approximately 59%, 35%, and 28% of survey respondents were supportive of the use of cyanide baiting, 1080 baiting, and humane toxins in fox eradication efforts on Tasmania, respectively. Comparatively, Farnworth et al. [70] found overwhelming acceptance of toxicant use in the control of non-native species to New Zealand by the general public and conservation groups. Furthermore, a study by Wilkinson and Priddel [71] found that the residents of Lord Howe Island were generally supportive of the use of toxicants in island wide rodent eradication efforts.

Stakeholders were in agreement that water contamination, human health impact, and incorrect usage of a toxicant were top concerns for any toxicant used in wild pig management. Additionally, toxicant impact on non-target species, legal liability for non-target damage, and soil contamination were all indicated as areas of increased concern. All of the stated concerns indicate that stakeholders are apprehensive about the overall safety of any wild pig toxicant, not only for human safety but also environmental safety. These findings are consistent with previous research that found stakeholders were concerned about the impact of toxicant use on non-target species, as well as human and environmental health [60, 71–74]. In other words, stakeholders appear unwilling to sacrifice their natural resource or personal health for improved wild pig management. Additionally, stakeholders were concerned about where the legal liability would fall if negative impacts did occur from toxicant use and how that liability may differ between an individual using a toxicant responsibly and in accordance with the usage guidelines and an individual who may not. Interestingly, stakeholders seem to understand that people are fallible, and are thus concerned about individuals administering or misusing a toxicant and the impact such actions might have. These same concerns have been expressed by wildlife biologists, managers, and researchers, so it seems that all parties involved share similar, legitimate apprehensions. Stakeholders have valid concerns regarding toxicant use, and in order for managers and policy makers to make informed decisions about the potential future use of toxicants in wild pig management, those concerns must be acknowledged and addressed before any decisions are made.

The information provided regarding both toxicants in the survey was the best information available at the time of dissemination. Sodium nitrite is a novel toxicant in US wild pig management, and efficacy testing is still underway in preparation for registration submission to the EPA. After survey administration, new information became available that questioned the specificity and safety of sodium nitrite as a toxicant. Specifically, recent field trials in the US have demonstrated that several non-target species are affected by the toxicant, including white-crowned sparrows (*Zonotrichia leucophrys*) and red-winged blackbirds (*Agelaius phoeniceus*). Notably, these non-target results were from the first and only field test of the initial prototype of a sodium nitrite bait in the US and therefore may not be representative of the final sodium nitrite bait product. Modifications to the bait are being made to further reduce impact on non-target species [75].

Had this information been available to present, stakeholder acceptability for sodium nitrite would most likely have been lower. What is important to note is that general toxicant use was not found to be overly unacceptable by any one stakeholder group. Thus, stakeholders may be relatively accepting of the idea of toxicants for wild pig management so long as their concerns regarding toxicant use are addressed and they are informed about the toxicants in question. However, following sodium nitrite reformulation and efficacy tests, further survey work may be warranted to ensure stakeholder support.

Purchasing and use regulations are one commonly used method for controlling who has access to a toxicant and how it is used [76, 77]. All stakeholder groups showed overwhelming support for the various purchasing and use regulations presented in the survey. Support for requiring that a wild pig specific bait dispenser and a toxicant be sold together to reduce impact on non-target species and requiring the completion of a toxicant use online training course to receive a purchasing and use permit reaffirms that stakeholders want a toxicant to be safe and they want it used correctly to reduce any potential for negative environmental or human health impact. Since the 1960’s public concern regarding toxicant use on human health and the environment and proper regulation has been growing [77–79], therefore, it is unsurprising that stakeholders would be supportive of wild pig toxicant regulations as well.

Distrust of government involvement in private property rights is a common issue for many landowners [54, 80]. Because of this innate distrust, it was surprising that there was a lack of resistance to a toxicant being unavailable to the public for use, and only allowing agency personnel to access and legally use any toxicant for wild pig management. Stakeholders would prefer to be able to buy and use a toxicant on their land. However, the lack of complete resistance to the possibility of only agency personnel or licensed applicators being granted legal access and use of a toxicant indicates that with some outreach and public education, managers and policy makers may receive little push back. Only allowing agency personnel or licensed applicators to apply a wild pig toxicant is the best way to safeguard proper use of a toxicant and minimize undesired impact, much like other commonly used pesticides. Additionally, liability would most likely fall on the licensed applicator, agency, or toxicant company if any unforeseen negative consequences of toxicant use happen to arise and not on the landowner.

Several limitations did occur during the project. Within the first week of disseminating the survey, the Auburn University main servers experienced a fire, causing the servers to shut down and all associated Auburn networks to go offline for approximately 1 day, including Qualtrics. During this time survey respondents were unable to access the survey. Once Qualtrics was back online, an email was sent out to all potential survey respondents explaining the technical difficulty, encouraging potential respondents to try again and apologizing for the inconvenience. Unfortunately, testing for non-response bias was not possible based on the invitation and data collection requirements of both the software and human subjects’ requirements. Specifically, IP addresses of respondents were not collected in order to protect respondent anonymity in accordance with the Auburn University IRB. Therefore, we were unable to use that information for non-response bias testing. Additionally, because ALFA and AFOA did not want to release the contact information of their members, each organization sent the survey link in an email directly to their members. Again, because we lacked access to member email addresses in addition to IP addresses, there was no way to identify specifically who participated in the survey and who did not. Therefore non-response bias testing was not possible. Due to the low response rate and inability to test for non-response bias, we are unable to claim these findings to be representative of each stakeholder group. However, these findings do help us to begin to develop a baseline understanding to assist future research into the topic. Lastly, since the survey was administered, new information has become available relating to the specificity of sodium nitrite in relation to non-target impact and water contamination. Such information puts into question the description of sodium nitrite presented to survey participants and therefore participant responses. Because of this new information, further study is warranted to assess how the most recent information regarding stakeholder perspectives on the use of sodium nitrite in wild pig management may differ.

## Conclusion

The findings here are the first attempts to begin understanding the social component of wild pig management through the use of toxicants. The results add to our current understanding of stakeholder’s perspectives towards wild pigs, while improving our understanding of future wild pig management, and the use of toxicants. Across the three stakeholder groups there seems to be minimal conflict between them regarding attitudes towards wild pigs, management objectives, and desired future population trends. Toxicant use in particular raises a variety of serious environmental, and human health concerns with all stakeholders, but as long as a toxicant is safe and proper use is regulated, stakeholders may be accepting and supportive of its use in wild pig management. By understanding and incorporating the above mentioned social factors with our current thorough understanding of the economic [38, 81–83] and environmental impact [81–83] of wild pigs, managers and decision makers gain a holistic understanding of the issue and are able to proceed towards a management solution that has a much higher probability for sustained success [38, 83, 84].

If any wild pig toxicant were to be legalized in the Southeast for agency personnel or licensed professionals only, gaining public support will be key for collaboration in efforts to remove wild pigs from the landscape. Because much of the land in the Southeast is privately owned, any hopes to reduce state or region wide wild pig populations, agencies will have to collaborate with private landowners to gain access to their land [85] and remove wild pigs. Without landowner support, agency personnel will only be able to utilize the toxicant on state or federally owned and managed lands. Furthermore, even if private lands are accessible, it is unlikely that complete eradication of wild pigs from the state will occur using toxicants as some individuals do enjoy hunting wild pigs for food or cultural purposes or are unwilling to put forth the time, money, or effort required to remove wild pigs from their property.

Toxicants may offer an additional tool that could drastically reduce wild pig densities, and subsequently the negative impacts associated with the species. Because wild pigs are a species of global concern [86], if toxicants were successful in safely reducing wild pig populations and impact in the US, application on an international level could have sizable positive impacts. Due to the limitations of this project, our findings should be viewed as a scoping investigation. We recommend that future research should use our findings as a baseline to delve further into the topics discussed with more representative samples. Specifically in regards to the acceptability of sodium nitrite and warfarin use in wild pig management, a repeat study is warranted.

## Supporting information

S1 Appendix Survey questions related to wild pig management and toxicant use amongst Alabama stakeholders. (DOCX)

S1 File (XLSX)
