# Supplementary material for: Stakeholder perspectives towards the use of toxicants for managing wild pigs
Source: PLoS One. 2021 Feb 5;16(2):e0246457. doi: 10.1371/journal.pone.0246457 (PMC7870098; doi:10.1371/journal.pone.0246457)
Supplement: S1 Appendix — (DOCX) [file pone.0246457.s001.docx]

S1 Appendix. Survey questions related to wild pig management and toxicant use amongst Alabama stakeholders.

1. What is your general attitude towards wild pigs? I dislike them a lot (1); I dislike them (2); I somewhat dislike them (3); I am neutral towards them (4); I somewhat like them (5); I like them (6); I like them a lot (7).
2. Complete the following sentence. In the future, you would like to see Alabama wild pig populations …Completely eradicated (1); Decreased drastically (2); Decrease moderately (3); Decrease slightly (4); Stay the same (5); Increase slightly (6); Increase moderately (7); Increase drastically (8).
3. How important is it to you that a management plan be developed by the Alabama Department of Conservation and Natural Resources to meet your preferred wild pig population trend as stated in the previous questions? Extremely unimportant (1); Slightly unimportant (2); Neutral (3); Slightly important (4); Extremely important (5).
4. Description of potential wild pig toxicant: **Name of the chemical compound:** Sodium nitrite **Common uses:** Meat preservative **Wild pig specific:** Yes, wild pigs lack necessary enzyme to reverse the effects of the toxicant **Mortality rate:** Approximately 95% in pen trials **Time of death:** Within 4 hours of bait consumption **Symptoms occurring leading to death:** 20-30 mins of labored breathing, loss of control of bodily movements, unconsciousness then death **Cause of death:** Lack of oxygen reaching brain and vital organs **Meat:** Safe for human consumption after death, excluding stomach contents. **Non-target species impact:** Concerns surrounding black bears accessing the toxicant. **Aquatic impact:** Insoluble in water, minimal threat to aquatic organisms **Scavenger impact (e.g., vultures):** Minimal concern. Please indicate how acceptable it would be to you if sodium nitrite was used to control wild pig populations in Alabama. Completely unacceptable (1); Somewhat unacceptable (2); Neutral (3); Somewhat acceptable (4); Completely acceptable (5).
5. Description of potential wild pig toxicant: **Name of the chemical compound:** Warfarin **Common uses:** Blood thinning medication and rodenticide **Wild pig specific:** Yes, due to low concentrations, wild pigs are unique in that they have the ability to consume the necessary quantities of bait over the required period of time to cause death (e.g., a 44 lb. dog would need to consume 13.2 lbs. of bait a day for 5 days to be fatal) **Mortality rate:** Approximately 98% in pen trials **Time of death:** With daily exposure and adequate amounts of bait consumed, death occurs after approximately 5 days.  **Symptoms occurring leading to death:** Lethargy, slowing of movement, bleeding then death **Cause of death:** Internal bleeding **Meat:** Not safe for human consumption, fat tissue is dyed blue to alert people to the contamination **Non-target species impact:** Concerns surrounding black bears accessing the toxicant and exposure to other predatory animals **Aquatic impact:** May be toxic to fish, do not apply toxicant directly in water or in areas with surface water or flooding potential **Scavenger impact (e.g., vultures):** May be toxic. Please indicate how acceptable it would be to you if Warfarin were used to control wild pig populations in Alabama. Completely unacceptable (1); Somewhat unacceptable (2); Neutral (3); Somewhat acceptable (4); Completely acceptable (5).
6. Questions 6 through 10 are all in relation to the following statement: If a wild pig toxicant were to be legalized in Alabama, please indicate your level of support for the following purchasing and use regulations. Must be 19 years of age or older to purchase a toxicant. Do not support at all (1); Somewhat do not support (2); Neutral (3); Somewhat support (4); Completely support (5).
7. Toxic bait and wild pig specific bait dispenser are required to be sold together to reduce exposure to non-target species (e.g., black bears). Do not support at all (1); Somewhat do not support (2); Neutral (3); Somewhat support (4); Completely support (5).
8. Toxicant is only sold by licensed vendors. Do not support at all (1); Somewhat do not support (2); Neutral (3); Somewhat support (4); Completely support (5).
9. A use permit obtained by completing an online training in toxicant application and safety is required to purchase toxicant. Do not support at all (1); Somewhat do not support (2); Neutral (3); Somewhat support (4); Completely support (5).
10. Toxicant is not available to the public. Only trained and licensed agency personnel have access to the toxicant and are legally allowed to use it. Do not support at all (1); Somewhat do not support (2); Neutral (3); Somewhat support (4); Completely support (5).
11. Questions 11 through 23 are all in relation to the following statement: Please select the option that represents the extent to which you are concerned about the following topics in relation to ANY toxicant use as a method of wild pig population control in Alabama. Humaneness of the toxicant. Totally unconcerned (1); Somewhat unconcerned (2); Neutral (3); Somewhat concerned (4); Extremely concerned (5).
12. Impact on non-target species (e.g., livestock, black bear, raccoon). Totally unconcerned (1); Somewhat unconcerned (2); Neutral (3); Somewhat concerned (4); Extremely concerned (5).
13. Personal time requirement. Totally unconcerned (1); Somewhat unconcerned (2); Neutral (3); Somewhat concerned (4); Extremely concerned (5).
14. Personal financial cost. Totally unconcerned (1); Somewhat unconcerned (2); Neutral (3); Somewhat concerned (4); Extremely concerned (5).
15. Eradicating wild pigs entirely. Totally unconcerned (1); Somewhat unconcerned (2); Neutral (3); Somewhat concerned (4); Extremely concerned (5).
16. Accidental water contamination. Totally unconcerned (1); Somewhat unconcerned (2); Neutral (3); Somewhat concerned (4); Extremely concerned (5).
17. Soil contamination. Totally unconcerned (1); Somewhat unconcerned (2); Neutral (3); Somewhat concerned (4); Extremely concerned (5).
18. Human health impact. Totally unconcerned (1); Somewhat unconcerned (2); Neutral (3); Somewhat concerned (4); Extremely concerned (5).
19. Ability to regulate use of the toxicant. Totally unconcerned (1); Somewhat unconcerned (2); Neutral (3); Somewhat concerned (4); Extremely concerned (5).
20. Incorrect usage of the toxicant. Totally unconcerned (1); Somewhat unconcerned (2); Neutral (3); Somewhat concerned (4); Extremely concerned (5).
21. Legal liability for non-target damage (e.g., accidental death of other animals due to toxicant) (11)
22. Effectiveness of the toxicant. Totally unconcerned (1); Somewhat unconcerned (2); Neutral (3); Somewhat concerned (4); Extremely concerned (5).
23. Public opinion. Totally unconcerned (1); Somewhat unconcerned (2); Neutral (3); Somewhat concerned (4); Extremely concerned (5).
24. Questions 24 through 34 are all in relation to the following statement: Please indicate the level of priority you would assign to the following **hypothetical** Alabama wild pig management objectives. Reduce wild pig damage. Very low priority (1); Low priority (2); Somewhat low priority (3); Neutral (4); Somewhat high priority (5); High priority (6); Very high priority (7).
25. Increase wild pig populations in the state. Very low priority (1); Low priority (2); Somewhat low priority (3); Neutral (4); Somewhat high priority (5); High priority (6); Very high priority (7).
26. Decrease wild pig populations in the state. Very low priority (1); Low priority (2); Somewhat low priority (3); Neutral (4); Somewhat high priority (5); High priority (6); Very high priority (7).
27. Stronger enforcement of current wild pig regulation and policy. Very low priority (1); Low priority (2); Somewhat low priority (3); Neutral (4); Somewhat high priority (5); High priority (6); Very high priority (7).
28. Restore damaged ecosystems. Very low priority (1); Low priority (2); Somewhat low priority (3); Neutral (4); Somewhat high priority (5); High priority (6); Very high priority (7).
29. Create wild pig management cooperatives to reduce individual costs and labor demands in order to remove wild pigs from larger areas of land. Very low priority (1); Low priority (2); Somewhat low priority (3); Neutral (4); Somewhat high priority (5); High priority (6); Very high priority (7).
30. Increase research to develop more cost and time effective wild pig control strategies. Very low priority (1); Low priority (2); Somewhat low priority (3); Neutral (4); Somewhat high priority (5); High priority (6); Very high priority (7).
31. Create a financial assistance program that aims to compensate individuals for economic loss associated with wild pig damage. Very low priority (1); Low priority (2); Somewhat low priority (3); Neutral (4); Somewhat high priority (5); High priority (6); Very high priority (7).
32. Increase funding to better facilitate state management. Very low priority (1); Low priority (2); Somewhat low priority (3); Neutral (4); Somewhat high priority (5); High priority (6); Very high priority (7).
33. Make high tech equipment (e.g., cell phone monitored trapping equipment) available for rent to land owners at a reasonable cost. Very low priority (1); Low priority (2); Somewhat low priority (3); Neutral (4); Somewhat high priority (5); High priority (6); Very high priority (7).
34. Make recreational wild pig hunting illegal. Very low priority (1); Low priority (2); Somewhat low priority (3); Neutral (4); Somewhat high priority (5); High priority (6); Very high priority (7).
35. What year were you born? (fill in the blank) 19___.
36. What is your gender? Male (1); Female (2); Other (3).
37. What is your highest completed level of education? Some high school (1); High school/GED (2); Some college, but no degree (3); Vocational/professional certification (4); Associates degree (5); Bachelor's degree (6); Master's degree (7); Doctorate (8).
38. What is your ethnicity? African American (1); Caucasian (2); Chinese (3); Japanese (4); Latino (5); Native American (6); Other Asian (7); Pacific Islander (8); Other (please specify) (9).
39. What is your total household income before taxes last year? Less than $14,999 (1); $15,000-$19,999 (2); $20,000-$24,999 (3); $25,000-$34,999 (4); $35,000-$49,999 (5); $50,000-$74,999 (6); $75,000-$99,999 (7); $100,000-$149,999 (8); $150,000 or more (9).
40. How long have you lived in Alabama? I have lived in Alabama for ____ year(s) (fill in the blank) (1); I live out of state, but I own/lease land in Alabama (2); I do not live or own/lease land in Alabama (3).
41. In what type of community do you currently live? Town/city with many neighbors (1); Outside a town with scattered neighbors (2); Rural area with few neighbors (3).
42. Do you own land in Alabama? Yes (1); No (2).
43. Approximately how much total land do you own? (acres).
44. In general, what is the primary purpose of your land? Farming (1); Hunting (2); Leasing (3); Forest products/timber (4); Residential (5); Other (please specify) (6).
45. On average, how often do you visit your land? I live on my property (1); Once a week (2); Once a month (3); Few times a year (4); Less than once a year (5).
